# Notes of 100 Consecutive Successful Operations from over 2,000 Aggregate Labours

**Published:** 1905-02-04

**Authors:** Glynn Whittle

**Affiliations:** Consulting Physician to the Liverpool Lying-in Hospital


### Hospital Clinics.

### NOTES OF 100 CONSECUTIVE SUCCESSFUL OPERATIONS FROM OVER 2,000 AGGREGATE LABOURS.

By Glynn Whittle, M.A., M.D. Cantab., M.R.C.P., Consulting Physician to the Liverpool Lying-in Hospital.

This series, commencing in the early years of my practice, now more than 20 years ago, consisted of cases with results uniformly favourable to the mothers. As the aggregate of the labours which yielded them, drawn partly from private and consulting practice, partly from the outdoor Ladies' Charity, and partly from the Lying-in Hospital, numbered more than 2,000, this gives the percentage, indicating operative intervention, as less than five per cent. In every doubtful case, in which, after contemplating the use of instruments, I finally decided against them and left the case to nature, both mother and child were saved. Enthusiasts for the frequent use of the forceps can therefore not sustain against me a charge of resorting too seldom to instrumental assistance.

The operations included one for the relief of jammed breech, one of detachment of adherent placenta from within an hour-glass contracted uterus, seven turning, and 91 forceps cases. In two of the latter the placenta was also adherent and had to be removed. In one case the hand presented, but further examination revealed the head, and manipulation ultimately effected a cephalic version. To prevent slipping away, which occurs sometimes in such cases, followed by the reappearance of the hand, the long forceps were applied, and the head secured and brought down.

In one case the cause of operation was hysteria, and in one prolapsus funis, the child being fortunately saved. The other main conditions which necessitated operation were hæmorrhage, contracted pelvis, rigidity, protracted labour and exhaustion, occipito-posterior positions of head, arm-presentations, and indirect complications, especially renal and chest affections.

Although many of the contracted pelves included difficult deformities, perforation was in no case necessary. The most valuable instruments for dealing with these extreme cases were the very long German forceps, which are less curved than most similar British instruments, and are of remarkable strength, a most admirable feature, weak instruments being in the highest degree objectionable and reducing the operator's efforts to a condition of struggling feebleness. In a very severe forceps operation it is necessary to give the patient adequate remissions between the long-sustained and oft-renewed efforts, or the bruising may lead to sloughing and occasion fistula. I have never found the effects of trauma to be more than temporary, causing inconvenience for a few days.

It was impossible to save all the children. The few who, owing to pelvic deformity of the mothers, could not survive delivery by forceps, would beyond doubt have been saved by Cæsarean section; but where are the woman and her husband—when it is candidly explained to the pair how comparatively safe to the mother is delivery by the natural passages —who would give their consent to an abdominal operation?

Among the cases undertaken on account of hæmorrhage the following three are of exceptional interest:—

Case 1. *Concealed Accidental Hæmorrhage.*—I was called urgently to this lady, a multipara, who had been discovered lying insensible on the floor a fortnight before her expected confinement. She was found by me lying on a sofa in her bedroom, greatly blanched, and very cold, pulse very feeble, barely 60, and faltering. Hot whisky, the only spirit available, was immediately administered, the patient placed in bed, and the clothing partly removed; pressure was applied to the uterus, and friction to the lower extremities. As soon as possible I proceeded to examine; the abdomen was unusually swollen and tender; no blood had escaped externally, and there was none in the vagina; the os uteri was high and closed, but did not resist the pressure of the tip of the finger, which on withdrawal was found, as expected, to be stained with blood. As the head could just be felt, I ruptured the membranes, liquor amnii and blood escaping through the vagina. There was no difficulty in dilating the os to a size which I deemed to be just sufficient to enable the forceps to be introduced, but beyond this the os would not expand. On attempting to introduce the fingers, the os uteri, which was very high up, receded from them; while in a similar manner the child's head receded from the os uteri, these causes preventing the application of the first blade in the usual way. The forceps were therefore applied in the following manner:—The first blade was passed up to and through the os uteri and on till it touched the head, like a probe touching a bone. The instrument was then used as a lever, with the fulcrum in the centre, the blade being the weight and the handle the power. The handle was pushed to the patient's right, a movement upwards and slightly in front of the axis of the uterus being executed at the same time, the blade thus gliding into its position alongside of the head. As it was now in a position hopelessly high up, owing to the receding of the head, it was then lowered, the lateral pressure on the handle being reversed, *i.e.* directed to the patient's left. In carrying out this movement, considerable and very steady lateral force was required to enable the blade to press on, and retain its grip of, the cranium. To prevent the head from again receding, I directed the nurse to hold the handle firmly, which thus enabled me to pass the second blade in the usual way. As soon as the blades were locked, two fluid drachms of liquid extract of ergot were given, but it was with the greatest difficulty that the patient could be sufficiently roused to swallow the draught. After this it took 45 minutes to deliver the child; to bring the head into the pelvis, and to check the hæmorrhage, traction from the shoulder was necessary, and had to be steadily maintained. The child was still-born, having evidently perished from the profuse internal hæmorrhage, which had also so jeopardised the life of its mother. Before delivery the completely relaxed condition of the uterus had become changed into that of a mild tonic contraction, probably the effect of the ergot, but not sufficient to materially help in expelling the child, while there was neither any spontaneous effort at normal uterine contraction, nor reflex action of the abdominal muscles to assist the instrumental delivery, so extreme was the degree of the exhaustion of the patient, who was quite collapsed for some time before the birth of the child. Following up the delivery with immediate compression of the uterus, its remaining contents were rapidly discharged with astonishing force and effect. A number of immense clots were successively shot, some on to the edge of the bed, and some even on to the floor beyond. The placenta came easily away, and the amount of liquid blood which escaped was but trifling. I washed out the uterus with hot water and the patient made a good, but necessarily tardy, recovery.

The length of the forceps used in this operation was almost 15 inches. Shorter instruments would not have availed, but I have frequently seen shorter instruments in ordinary use as long forceps. The resources of a practitioner thus equipped for a similar case would necessarily be reduced to turning, an alternative which would certainly diminish and probably destroy the collapsed patient's chance of surviving her delivery.

Case 2. *Partial Placenta Prævia.*—This was a case in the Lying-in Hospital. The hæmorrhage, which was free when the pains set in, could not be controlled by plugging, rupturing the membranes, or separating the part of the placenta, which adhered within the orificial zone. I, therefore, with three fingers endeavoured to keep up the placenta, while it was hoped that the pains, assisted by pressure applied to the uterus through the abdominal wall, would enable the head to engage the brim. This manœuvre, which is an easy one and generally successful, failed in this case. More of the placenta appeared, with a continuance of the hæmorrhage. The condition described in the previous case—the receding of the cervix from the fingers and of the head from the cervix—was also present here in a marked degree. I therefore passed the first blade of the forceps as in the previous case, but in attempting to lower it in the manner described, so as to bring the lock outside the vulva, it failed to retain its grasp of the head. The failure of a second attempt convinced me of the uselessness of trying to persevere with the forceps. Meanwhile, the pains and hæmorrhage showed no signs of abating, I therefore decided to turn, and, the patient having been placed under the influence of chloroform by one of my colleagues, proceeded to do so.

Having introduced the hand into the vagina, I found that the cervix did not long resist the four fingers and thumb and soon allowed the knuckles to slip within its circle. Knowing the child to be with its back forwards I endeavoured to reach the feet by passing my hand along the posterior wall of the uterus, but, in spite of the chloroform, this attempt invariably excited such reflex uterine contraction that, for fear of rupture, it had to be abandoned. Explorations in the transverse diameter were, of course, not practicable, for, if they had been possible, which is most unlikely, they would have brought me to the shoulders and arms, thus embarrassing my progress upwards. So I passed my hand to the front, beginning by adapting the concavity of the palm to the convexity of the occiput. Gradually raising my hand, I kept to the middle line, or nearly so, till my wrist was curved on the occiput, and my hand would thus be clear of the left shoulder. Then a lateral movement at the left of the uterus and round the left of the child's body, sweeping past the arm and hand, brought me to a foot, which I brought down without much difficulty.

The success which here followed the approach described is in marked contrast to the condemnation at times expressed of the passage of the operator's hand between the child's back and the mother's abdomen and round the child's body; but the chief justification of this objection appears to lie in the idea that the feet, which are placed towards the mother's spine, would be seized and dragged down over the child's back. The plan I had in view, however, and which I adopted, was, after seizing the foot, to continue the lateral passage of my arm, which had already been chiefly accomplished, and retire along the middle line of the back, as I had advanced along the middle line of the front. In carrying out the various stages of the proceeding I encountered no serious interruption. The child being a fine one, and well filling the brim, gentle traction was quite sufficient to press on the placenta and control the hæmorrhage.

Case 3.—*Central Placenta Prævia.* This was a patient admitted into the Lying-in Hospital with a recent history of hæmorrhage. On examination the cervix was very high up, thick and trumpet-shaped. The anterior lip was three-quarters of an inch in length, the posterior lip one inch and a half, the os internum being firmly closed. As neither pains nor any signs of hæmorrhage were present, she was ordered to be kept in bed and closely watched. About 12 hours afterwards I received an alarming message that the patient was bleeding to death. On arrival I found the hæmorrhage temporarily abating, the matron having followed my instructions to plug the cervix with one or more of her fingers, should occasion arise, pending my arrival. Examining, I found the placenta centrally attached, but only the index finger could be introduced through the os. As the hæmorrhage soon reappeared and pains set in, plugging was out of the question, and I decided to dilate with my hand. This was slowly accomplished in the old-fashioned manner, and, as soon as practicable, that part of the placenta which adhered to the lower zone was separated, but without arrest of the hæmorrhage. Palpation revealed the child to be head downwards and back forwards, so I continued the expanding process till my hand was able to enter the uterus. I then turned in the usual manner and safely delivered the mother, but the child was still-born. Throughout the operation I had had to contend with undue and persistent rigidity of the soft parts, constituting no slight element of danger, and necessitating the utmost caution. The entire proceeding of dilating the cervix and delivery by turning, which was continued uninterruptedly from its commencement, was a fatiguing operation, and occupied two hours and a half.

In conclusion, I would once more emphasise the great lesson which experience of hæmorrhage before delivery ever brings us, viz., to stand very closely by our patient. We must watch her practically all the time, till the danger is over, or we must take active steps to effect immediate delivery. When in doubt, I have always followed the latter course, and it is the safest rule.

